# Zoledronate combined metal-organic frameworks for bone-targeting and drugs deliveries

**DOI:** 10.1038/s41598-022-15941-w

**Published:** 2022-07-19

**Authors:** Yixiao Pan, Jiahao Wang, Zichao Jiang, Qi Guo, Zhen Zhang, Jingyi Li, Yihe Hu, Long Wang

**Affiliations:** 1 Department of Orthopedics, Xiangya Hospital, Central South University, Changsha, China; 2 Department of Orthopedics, First Affiliated Hospital, School of Medicine, Zhejiang University, Hangzhou, China; 3 Hunan Engineering Research Center of Biomedical Metal and Ceramic Implants, Xiangya Hospital, Central South University, Changsha, China; 4 National Clinical Research Center for Geriatric Disorders, Xiangya Hospital, Central South University, Changsha, China; 5 Hunan Key Laboratary of Aging Biology, Xiangya Hospital, Central South University, Changsha, China

**Keywords:** Nanomedicine, Nanoscale materials, Drug delivery

## Abstract

Medicine treatments for bone-related diseases such as osteoporosis, bone metastasis, osteomyelitis, and osteolysis are often limited by insufficient drug concentration at the lesion sites owing to the low perfusion of bone tissue. A carrier that can deliver multiple bone destruction site-targeting drugs is required to address this limitation. Here, we reported a novel bone-targeting nano-drug delivery platform formed by the integration of zoledronate (ZOL) and zeolitic imidazolate framework-8 (ZIF-8) nanoparticles. The ZOL mixed zeolitic imidazolate framework (ZZF) nanoparticles were synthesized in water at room temperature (25 °C), where many biomacromolecules could maintain their activity. This allowed the ZZF nanoparticles to adapt the encapsulation ability and pH response release property from ZIF-8 and the excellent bone targeting performance of ZOL simultaneously. Considering the ease of preparation and biomacromolecule-friendly drug delivery of this nano platform, it may be useful in treating bone-related diseases.

## Introduction

Osteoporosis, bone tumor, bone infection, and periprosthetic osteolysis are complicated illnesses in orthopedics. For these diseases, a combination of factors leads to systemic or local high bone turnover, thereby accelerating bone resorption and consequently causing bone deconstruction and reduced bone strength^1–3^. Although certain drugs can target these pathological changes, owing to insufficient blood supply, they might be metabolized before reaching an effective concentration at the lesion areas. Therefore, an efficient drug delivery platform targeting the bone resorption area is highly desirable.

The nanomedicine delivery platform has been the focal point of numerous recent biochemical research studies. Nanoparticles with metal-organic frameworks (MOFs) have received increasing attention as nanomedicine delivery platforms. Jarai et al. evaluated the UiO-66, a zirconium and terephthalic acid based MOF, as a novel platform which have promising drugs loading and pulmonary delivery capacities^4^. Zhuang et al. investigated the platelet membrane-coated MOF as a nanodelivery system loaded with siRNA can achieving gene silencing in vivo^5^. The variety of MOF provides new ideas for medicine treatments, and the most important features for bone tissue-related diseases should be remarkable biosafety, multiple-drug loading capacities, and high bone targeting efficiency. The targeting capacity of nanoparticles is increased by combining them with bone-targeted agents (BTA) such as aptamers^6,7^, and antibodies^8,9^ and small molecules^10,11^.

Zoledronate (ZOL), also known as zoledronic acid, is the third generation of bisphosphonate approved for the treatment of osteoporosis and bone metastases^12^, and exhibits promising bone targeting ability, particularly at the high bone turnover sites^13^. Our previous study showed that PLGA nanoparticles loaded with ZOL enhanced bone-targeting abilities^14^. Previous studies have explored many methods of incorporating ZOL into nanoparticles. Sun et al. reported that ZOL anchored onto the mesoporous silica nanoparticles can yield bone-targeting ability^10^, Qiao et al. reported that ZOL conjugated onto the mesoporous silica-coated upconversion nanoparticles can target the osteocytes to attenuate early breast cancer bone metastasis^15^. However, the methods of binding ZOL to nanoparticles discussed in these studies are considerably limited because they require the use of organic solvents such as DMF and DMSO and prolonged heating, which pose serious challenges for many biomacromolecules such as nucleic acids and proteins.

Considering the aforementioned factors, we focused on a promising nanoparticle, Zeolitic imidazolate framework-8 (ZIF-8), which is a member of ZIFs and consists of zinc ions and 2-methyl-imidazole (2-MIM). ZIF-8 was frequently employed as a nanocarrier for many bio-macromolecules owing to its low toxicity and remarkable biocompatibility^16–18^. Moreover, ZIF-8 can be synthesized in water at room temperature (25 °C) thereby allowing biomedical macromolecules to retain their activities when loaded^19–21^. We noted both 2-MIM and ZOL have an imidazole group. Based on this, we suspected that ZOL could possibly play a role in the synthesis process of ZIF-8 and form novel mixed ZOL-ligands zeolite frame (ZZF) nanoparticles which could simultaneously adapt the excellent carrier properties of ZIF-8 and the remarkable bone-targeting ability of ZOL.

**Figure 1:**
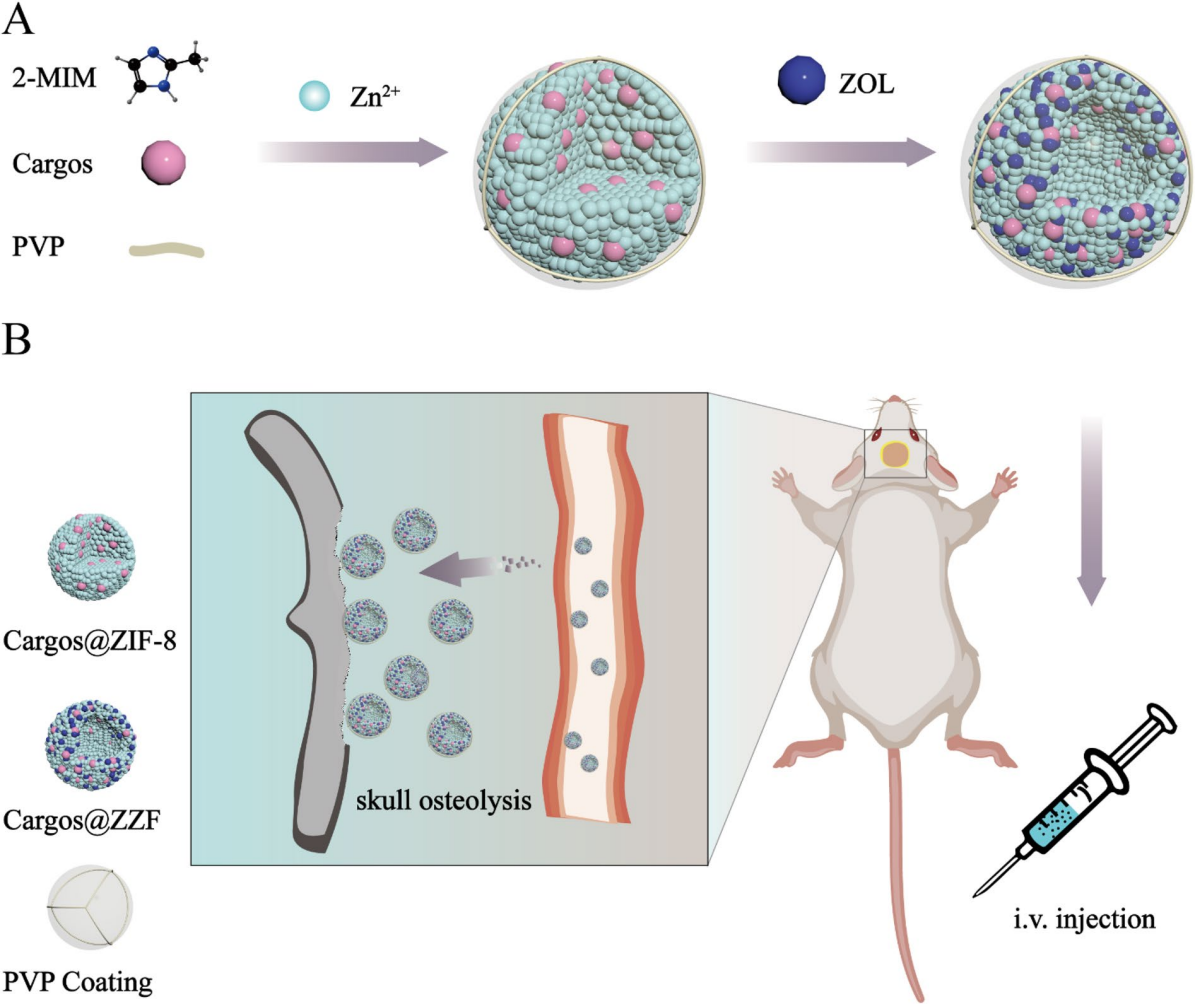
(**A**) Schematic illustration of the construction of the Cargos@ZZF nanoparticles and PVP coating. Cargos represent different substances such as doxorubicin, bovine serum albumin, and siRNA. (**B**) Schematic illustration demonstrating the osteolysis area targeting ability of the ZZF@PVP nanoparticles in the calvaria resorption mouse model.

Therefore, in this study, we constructed a bone-targeting drug delivery platform based on the ZIF-8 with mixed ZOL and 2-MIM ligands (ZZF) nanoparticles (Figure 1). We first investigated the various cargo loading abilities of the ZZF nanoparticles and their pH-sensitive release behaviors. The cellular internalization and toxicity were conducted in vitro, and the bone resorption area targeting the ability of ZZF was detected by fluorescence imaging using a mouse calvaria resorption model. This nanoplatform, which is easily synthesized, and exhibits biosafety and multiple-drug loading capacity bone-targeting features presents fascinating application prospects in bone resorption lesions.

## Results and discussion

### Synthesis and characterizations of ZZF@PVP nanoparticles

**Figure 2:**
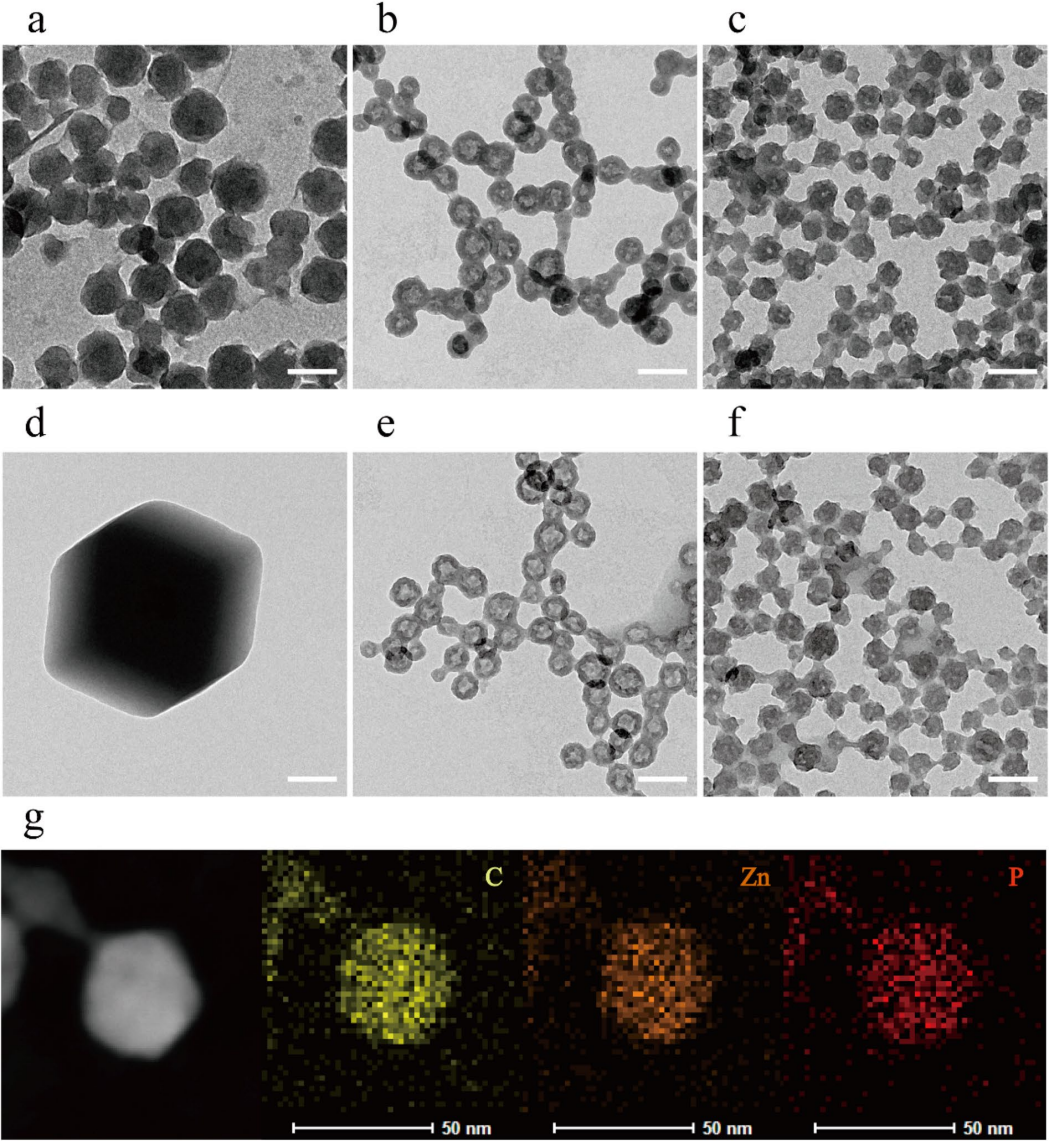
TEM images of ZIF-8, ZZF, and ZZF@PVP nanoparticles when synthesized (**a**–**c**) and after storage in water for 2 weeks (**d**–**f**), respectively. Scale bar = 100 nm. TEM and the element mapping image of the distribution of carbon, zinc, and phosphorus in individual ZZF@PVP nanoparticles (**g**). Scale bar = 50 nm.

The morphology of the ZIF-8 and ZZF@PVP nanoparticles was characterized by a transmission electron microscope (TEM). The ZIF-8 nanoparticles synthesized in aqueous were spherical with certain faceted particles being approximately 80 nm in size, which is an indication of the initial stages of crystallization of the ZIF-8 nanoparticles^22^. Following the addition of ZOL, ZZF nanoparticles exhibited a different hollow spherical shape and a reduction in size at approximately 50 nm, a suitable size for intracellular delivery. The ZZF@PVP is similar in size to ZZF with PVP molecules covered (Fig. 2a–c). These significant shape changes in ZZF might be due to the strong covalent binding ability of ZOL compared with that of 2-MIM. When ZOL binds to the surface of ZIF-8 nanoparticles, its relatively strong deprotonation ability reduces the pH value at the core of ZIF-8, leading to partial decomposition of ZIF-8 particles and then forming new hollow structures. Element mapping of C, Zn, and P also proved that ZOL was evenly distributed in nanoparticles (Fig. 2g).

Then we studied the stability of the nanoparticles in water. After storage in pure water for 2 weeks, the ZIF-8 nanoparticles formed significantly larger (1–2 μm) faceted crystals, while the ZZF and ZZF@PVP nanoparticles maintained the same size with slight changes in shape (Fig. 2d–f). These results indicate that ZIF-8 tended to form larger crystals in water, and the combination of ZOL with ZIF-8 can significantly improve the stability of nanoparticles in water, which is of great significance for biomedical applications.

**Figure 3:**
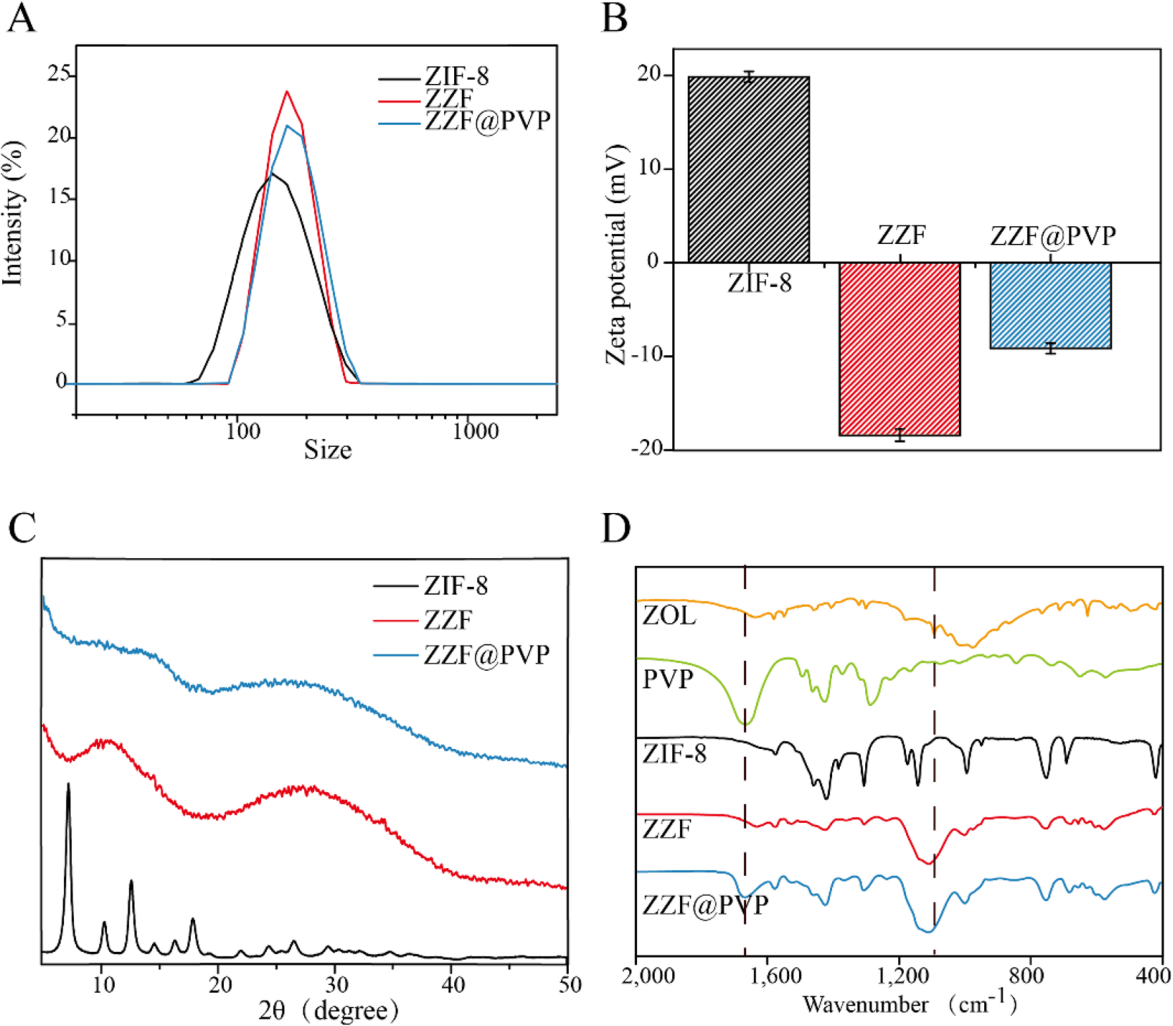
(**A**) DLS, (**B**) zeta-potential, and (**C**) powder XRD of ZIF-8, ZZF, and ZZF@PVP. (**D**) FTIR spectra of free ZOL, PVP, ZIF-8, ZZF, and ZZF@PVP.

According to the DLS measurement results (Fig. 3A), ZZF@PVP nanoparticles exhibit a decreased hydrodynamic diameter (117 nm) compared to ZIF-8 nanoparticles (145 nm). The surface zeta potential of the ZZF shifted from 20 to − 18.3 mV owing to the modification of the negative charged ZOL, and the loading of PVP can significantly reduce the zeta potential of ZZF@PVP to − 7 mV (Fig. 3B). Negatively charged nanoparticles are more stable in vivo environments, which is conducive to the accumulation in the target sites.

Powder X-ray diffraction spectra analysis confirmed that the ZIF-8 nanoparticles maintained a certain degree of crystallinity while the ZZF@PVP nanoparticles exhibit amorphous characteristics (Fig. 3C). This change is different from that in previous studies where ZIF-8 encapsulated protein or small molecule drugs retained the crystal property^16,18,23,24^, which also confirmed that zoledronate, as a ligand, was involved in the formation of such composite nanoparticles and destroyed the original crystal form of ZIF-8.

The formation of ZIF-8 and ZZF@PVP was further evidenced by the FTIR (Fig. 3D). The FTIR spectra revealed two characteristic intensities at approximately 1660 cm^−1^ of carbonyl (C=O) and 1100 cm^− 1^ of phosphoryl (P=O) demonstrating the efficient loading of PVP and ZOL, respectively.

The content of ZOL in the ZZF@PVP nanoparticles was 14.1 wt% by HPLC analysis.

### Drug loading and pH-controlled release

Drug loading capacity is an essential characteristic of drug loading nanoparticles. We used DOX, bovine serum albumin (BSA), and small interfering RNA (siRNA) as examples to investigate the loading capacity of the ZZF@PVP nanoparticles on small molecular drugs, proteins, and nucleic acids, respectively. After adjusting, the loading efficiency values for DOX, BSA, and siRNA were 6.6 wt%, 15.5 wt%, and 0.3 OD/mg (1 wt%), while the encapsulation rates were 90%, 93%, and 96%, respectively. Under these conditions, the nanoparticles loaded with these materials can still exhibit an acceptable size and stability in water. The loading capacities of different types of substances are similar to those of ZIF-8 in previous studies (The loading capacities for large pDNA molecules^16^, protein Cyt c^25^, and DOX^26^ of ZIF-8 was reported ≈ 2.5–3.4 wt%, 8 wt%, 19.7 wt%, respectively).

**Figure 4:**
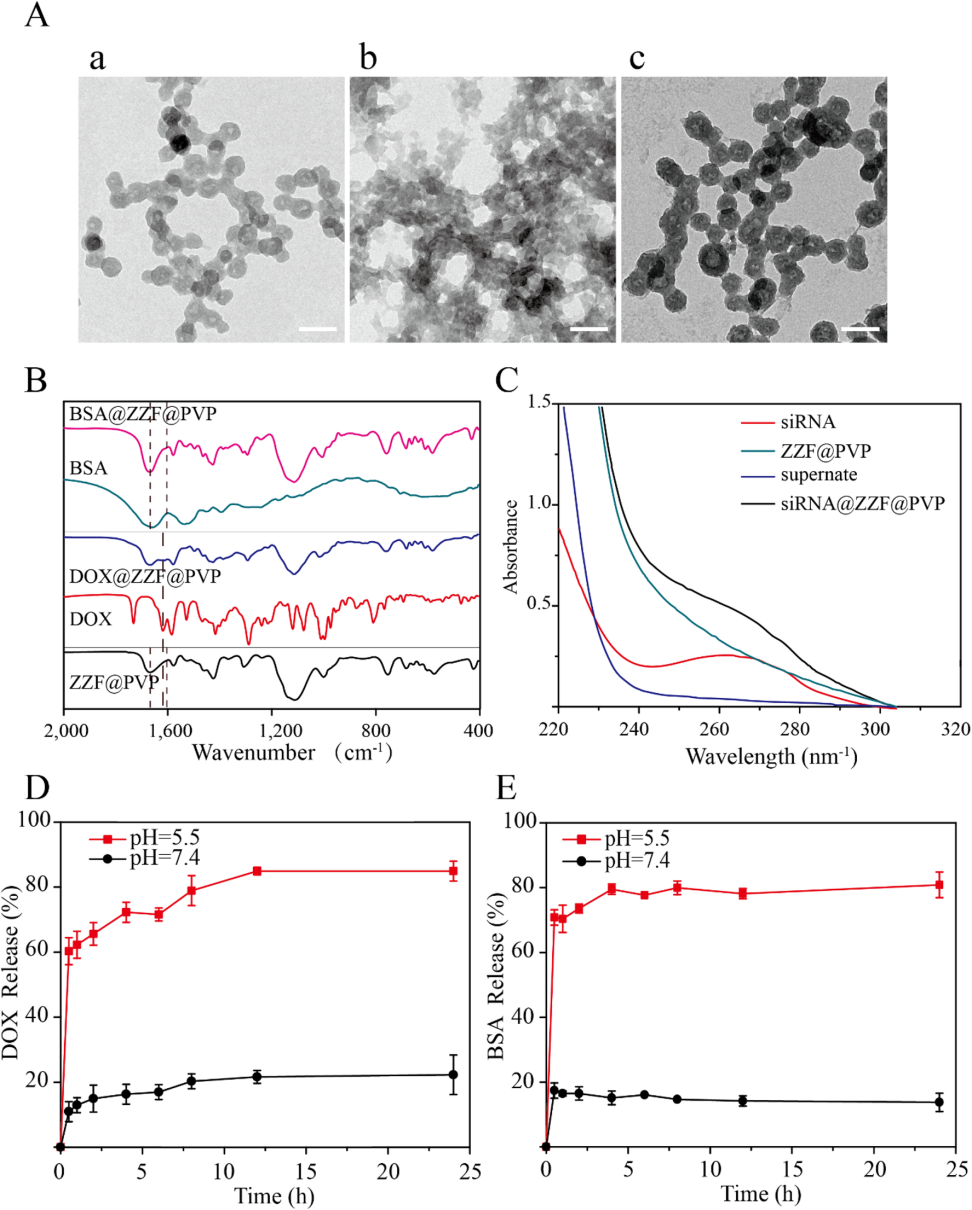
(**A**) TEM images of ZZF@PVP loaded with (a) DOX, (b) BSA, and (c) siRNA. Scale = 100 nm. (**B**) FTIR spectra of ZZF@PVP, free DOX, DOX@ZZF@PVP, free BSA, and BSA@ZZF@PVP. (**C**) UV–vis spectra of free siRNA, siRNA@ZZF@PVP, ZZF@PVP NPs, and the supernate of the as-synthesized siRNA@ZZF@PVP after centrifugation. (**D**,**E**) DOX and BSA release profiles for DOX and BSA loaded ZZF@PVP at pH 5.5 and 7.4.

TEM showed the slight changes in the size and morphology of nanoparticles loaded with DOX and siRNA while BSA-loaded nanoparticles exhibited decreased size and were aggregated (Fig. 4A). For drug-loaded nanoparticles, package size exhibited a slight change; particularly, the morphology of the nanoparticles package of the BSA was significant changed with a decreased size, while no significant particle sizes change was observed after the load of DOX and siRNA; they remained approximately 50 nm, which is the ideal size for drug-loading nanoparticles. The successful loading of DOX, BSA, and siRNA was evidenced by FTIR and UV–Vis spectrum. The characteristic peak (1670 cm^−1^) of benzene were observed in the FTIR spectrum of the DOX@ZZF@PVP nanoparticles, and the peaks (1598–1650 cm^−1^) of amide I band region was observed in the spectrum of the BSA@ZZF@PVP nanoparticles, which indicated that DOX and BSA were successfully loaded onto the ZZF@PVP nanoparticles, respectively (Fig. 4B). As shown in Fig. 4C, siRNA showed the adsorption peak at 260 nm while no characteristic adsorption peak was observed for ZZF@PVP. However, siRNA@ZZF@PVP nanoparticles clearly exhibited the characteristic peak of siRNA, suggesting that siRNA was encapsulated into the ZZF@PVP nanoparticles. Moreover, the supernate of the as-synthesized siRNA@ZZF@PVP has no characteristic adsorption peak, which indicated that the loading efficiency of siRNA on ZZF@PVP nanoparticles is quite satisfactory.

pH-release behaviors of the ZZF@PVP nanoparticles revealed that this nanoparticle can be stable at pH = 7.4 but disassembled quickly at pH = 5.5 (Fig. 4D,E). This pH-dependent dissociation property suggests that when applied in vivo, the ZZF@PVP nanoparticles can protect the drugs loaded until they reach the acid environment such as tumors, inflammation, bacterial infection sites, and intracellular lysosomes, allowing the drugs to reach a higher concentration in the target tissues or cells.

### Cytotoxicity assay

**Figure 5:**
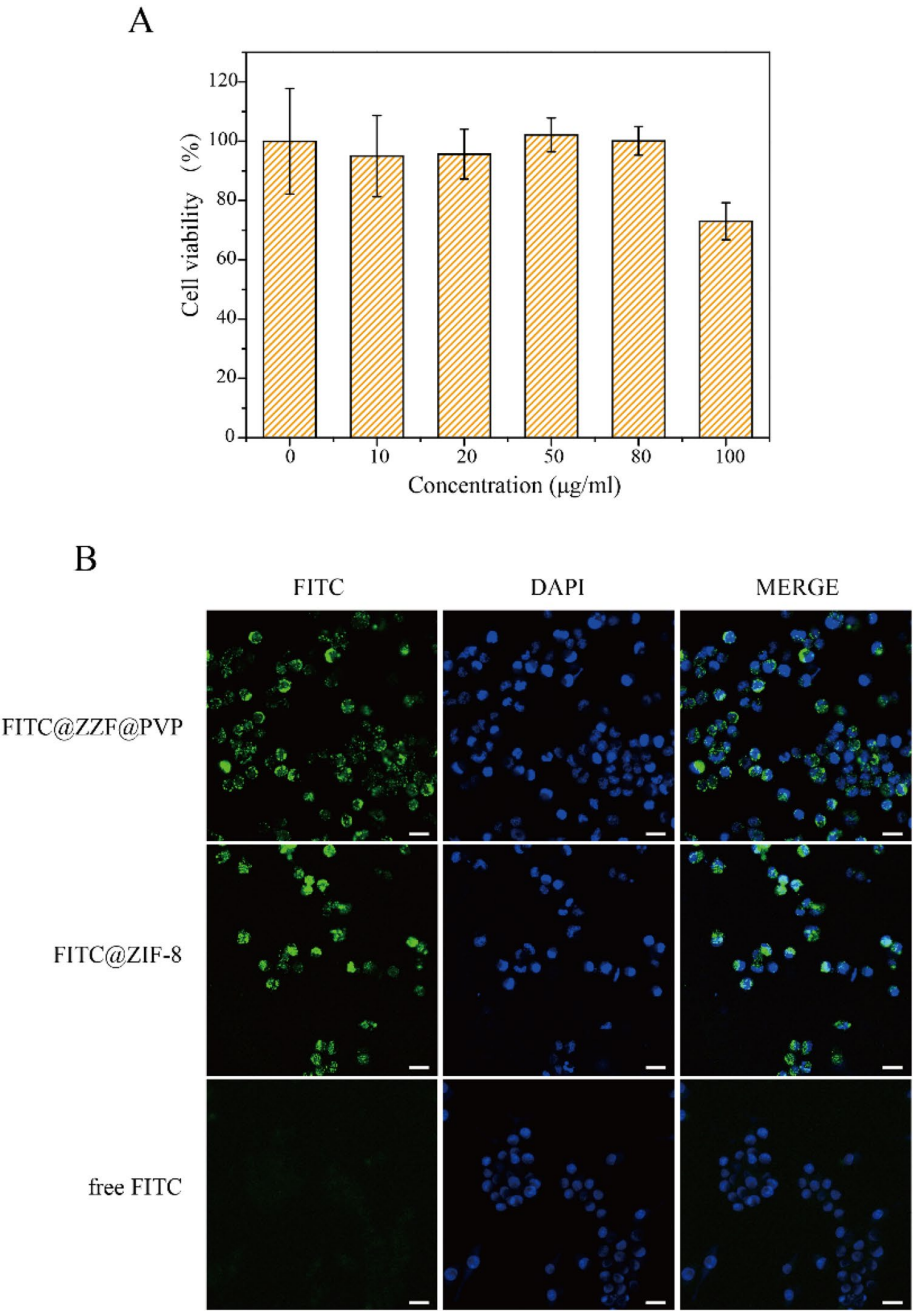
(**A**) Cytotoxicity assay of ZZF@PVP. The cell viability values (%) are determined by incubating RAW264.7 cells with ZZF@PVP NPs of varying concentrations (10, 20, 50, 80, 100 µg/ml) for 24 h. (**B**) Fluorescence images of RAW264.7 cells treated with FITC@ZZF@PVP, FITC@ZIF-8, and free FITC. Scale bar = 20 μm.

The effects of different concentrations of free nanoparticles on cell viability were studied by CCK-8 measurement on the RAW264.7 cell line. ZIF-8 and ZZF@PVP had a toxic effect on RAW264.7 cells in a dose-dependent manner. A low concentration of ZZF@PVP nanoparticles (< 80 μg/ml) had negligible toxic effect on RAW264.7 cells because the cell viabilities of both nanoparticles exceeded 95% at each concentration (Fig. 5A). This result is similar to the results of previous studies on ZIF-8^18,26^. In vitro cytotoxicity tests of ZIF-8 alone often show an inhibition effect on cells at high concentration, while low concentration shows no significant toxicity. In addition, we also tested the cytotoxicity of ZOL, and the results showed that ZOL had no obvious toxicity to RAW264.7 cells at the concentration of 14.1 μg/ml (Supplementary Fig. S2). Therefore, ZZF@PVP exhibits remarkable biocompatibility in vitro and is safe for biomedical applications.

### Cellular uptake of the ZZF@PVP nanoparticles

Next, we examined the intracellular ability of the nanoparticles. The tracer fluorescein FITC was encapsulated in the ZZF@PVP and ZIF-8 nanoparticles, and intracellular internalization was monitored by confocal laser scanning microscopy (CLSM). As shown in the Fig. 5B, the green fluorescence of cells treated with free FITC was negligible, indicating a low degree of cell uptake. In contrast, a strong green fluorescence signal was observed in cells treated with FITC@ZZF@PVP and ZIF-8 nanoparticles, indicating relatively high cell uptake. Previous studies have shown that while most molecules cannot be effectively internalized by cells, nanoparticles with sizes ranging from 50 to 200 nm can be actively incorporated into cells through different endocytic pathways^27^. As seen in the results above, the ZZF@PVP nanoparticles with a size of approximately 50 nm were easily integrated into cells.

### In vivo bone-targeting ability of the ZZF@PVP nanoparticles

**Figure 6:**
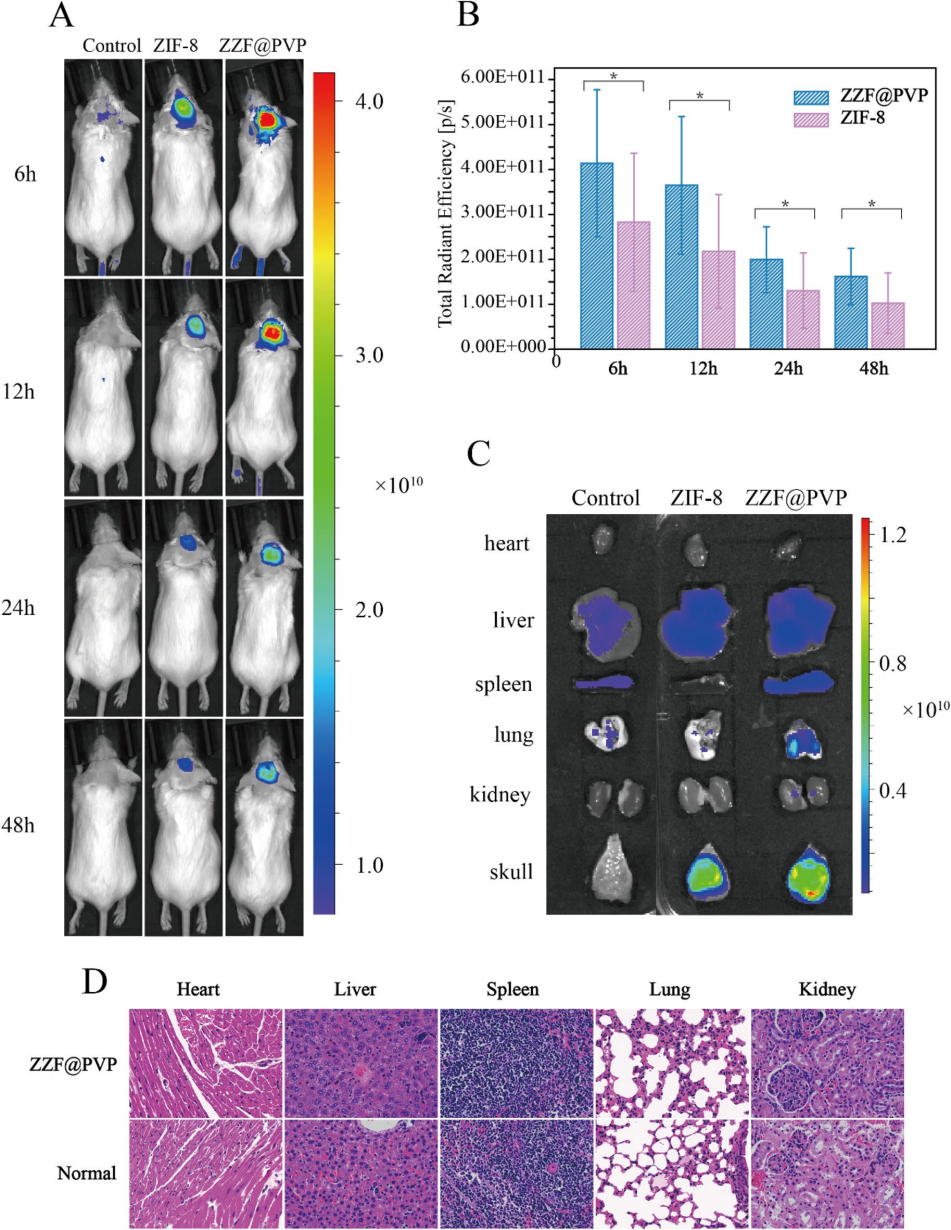
(**A**) Living fluorescence imaging of mice from control, ZIF-8, and ZZF@PVP groups at different time points after injection. (**B**) Total radiant efficiency analyses of the surgery area between the control, ZIF-8, and ZZF@PVP groups at 6, 12, 24, and 48 h after injection. (**C**) Representative fluorescence images of ex vivo heart, liver, spleen, lung, kidney, and skull of mice from the three groups at day 5 after intravenous injection. (**D**) H&E stain of major organs of ZZF@PVP group mice at day 5 after a single injection of nanoparticles.

We used the calvaria resorption mouse model to evaluate the bone targeting ability of the ZZF@PVP in vivo. Mouse were separated as three groups: the control group (shame surgery mouse), the ZIF-8 group, and the ZZF@PVP group. The fluorescence images were measured on the animal imaging system after a single injection through the tail vein of the ZIF-8 (for the ZIF-8 group) and ZZF@PVP (for the control group and ZZF@PVP group) nanoparticles containing the in vivo fluorescent tracer Cy5.5, respectively. Fluorescence images showed that the fluorescence signal of the ZZF@PVP group was significantly stronger than that of the ZIF-8 group in the operation area of mice in different time points, while there was almost no fluorescence signal in the sham control group injected with the same Cy5.5@ZZF@PVP nanoparticles as the ZZF@PVP group (Fig. 6A). The analyses of total radiant efficiency of the two groups also proved the ZZF@PVP nanoparticles significantly accumulated in the operation area (Fig. 6B). The fluorescence signal in the ZIF-8 group suggests that ZIF-8 nanoparticles could be accumulated in this PE particles induced inflammation area via the enhanced permeability and retention (EPR) effect, and only a few scattered fluorescence signals were captured in the sham control group. After comparing the results of the two groups above, we speculated that the concentration of ZZF@PVP may be achieved through a combination of the EPR effect and the targeting ability of ZOL to this PE particles induced high bone turnover sites. On the 5th day after injection, the mice were sacrificed, and the main organs and tissues including heart, liver, spleen, lung, kidney, and skull were collected for fluorescence imaging. Similar to the results of living images, the skull of the ZZF@PVP group exhibits excellent fluorescence signals (Fig. 6C). The fluorescence imaging results above confirmed that the ZZF@PVP nanoparticles had obvious targeting ability to the PE particles induced high bone turnover sites. Although the ZIF-8 nanoparticles also accumulated in this region to a certain extent owing to obvious inflammation in the osteolysis region, the targeting effect in the ZZF@PVP group was significantly more evident. This osteolysis region targeting ability means that the ZZF@PVP nanoparticles would deliver drugs more effectively to the area which has high bone turnover and destruction, such as osteoporosis, bone metastasis, osteomyelitis, and osteolysis, the future application of the scene is very broad.

H&E-stained histological analysis of the main tissues (heart, liver, spleen, lung, and kidney) indicated that the injection of ZZF@PVP nanoparticles did not cause acute organ damages (Fig. 6D).

## Methods

### Materials

Zinc nitrate hexahydrate [Zn (NO_3_)_2_·6H_2_O], 2-methylimidazole (2-MIM), sodium hydroxide, bovine serum albumin (BSA), and Doxorubicin (DOX) were obtained from Sigma-Aldrich (USA). Polyvinyl Pyrrolidone (PVP, MW ≈ 40 kD) was obtained from Adamas (Shanghai, China). Zoledronate (ZOL) was obtained from Energy Chemical (Shanghai, China). The triethylamine, methanol and phosphoric acid were purchased from Aladdin Biochemical (Shanghai, China). The sulfo-Cy5.5-M free acid and fluorescein isothiocyanate (FITC) were purchased from US Everbright (Suzhou, China). The siRNA was synthesized by GenePharma (Shanghai, China).

### Synthesis of the ZIF-8 and ZIF-8@PVP nanoparticles

The ZIF-8 and ZIF-8@PVP nanoparticles were synthesized in water as previously reported^26^ with some modifications. Briefly, 41 mg of 2-MIM and 120 mg PVP (only added for the synthesis of the ZIF-8@PVP) were added in 9 ml of water and stirred for 10 min. Afterwards, 1 ml of water containing 29.8 mg Zn (NO_3_)_2_·6H_2_O was added drop by drop. After stirring for 2 h, the ZIF-8 and ZIF-8@PVP nanoparticles were collected by centrifuged at 15,000×*g* for 20 min.

### Synthesis of the ZZF and ZZF@PVP nanoparticles

The ZZF and ZZF@PVP nanoparticles were synthesized based on the precursors ZIF-8 and ZIF-8@PVP. As the same procedures mentioned above, after Zn (NO_3_)_2_·6H_2_O aqueous solution was added to the 2-MIM and PVP mixed solution (PVP was added to form the ZZF@PVP nanoparticles) and stirring for 2 h, 0.6 ml aqueous solution containing 8.7 mg ZOL whose pH was adjusted to 7 with a sodium hydroxide solution was added. After stirring for an additional 12 h, the ZZF and ZZF@PVP nanoparticles were centrifuged at 15,000×*g* for 20 min and washed twice with water before being redisposed into the water for further use. The molar ratio of Zn to 2-MIM to ZOL was 10:50:3.

### Synthesis of DOX, BSA, and siRNA loaded ZZF@PVP nanoparticles

In accordance with the biomimetic mineralization procedures^20^, 2 mg BSA, 5 mg of DOX, and 5OD FAM-siRNA were dissolved into a mixture of 9 ml 2-MIM and PVP and stirred for 10 min; then Zn ion was added. The drug-loaded nanoparticles were formed using the aforementioned method. The loading efficiency (LE %) was calculated using the equation: LE (%) = (drugs initial − drugs residual)/weight of nanoparticles, and the encapsulation efficiency (EE %) was calculated using the equation: EE (%) = (drugs initial − drugs residual)/drugs initial. The residual amount of drugs refers to the concentration of drugs in the supernatant of nanoparticles after centrifugation, which was detected using the BCA kit (for BSA) and UV-Vis absorption (for DOX and siRNA).

### Nanoparticle characterization

The particle hydrodynamic diameter and zeta-potential were assessed by dynamic light scattering (DLS) using a Nano zeta sizer P2000 (Malvern, Worcestershire, UK). Transmission electron microscopy (TEM) was used to investigate the morphologies of the nanoparticles (FEI Tecnai G2 F270, Hillsboro, USA). Powder X-ray diffraction (XRD) patterns were recorded on the D8 Advance X-ray diffractometer (X ‘Pert Pro MPD, Holland). UV-Vis absorption spectra were measured with a UV-2400 spectrophotometer (Shimadzu, Japan). Fourier transform infrared spectroscopy (FTIR) was recorded on Nicolet iS20 (Thermo Scientific, USA). The content of ZOL in the ZZF and ZZF@PVP nanoparticles was determined by was analyzed with high-performance liquid chromatography (HPLC, LC-20AD, Shimadzu, Japan). HPLC analysis of ZOL was performed with a Shimadzu LC-20AD HPLC system and a Phenomenex Luna 5 μm C18 column. The column temperature was maintained at 37 °C. The mobile phase was a mixture of solvent A [Phosphate buffer (2 ml triethylamine, add water to 1000 ml, adjust pH to 3.0 with phosphoric acid)] and solvent B (methanol), with a ratio of 19:1 and a flow rate of 1 ml/min. The total analysis time was 20 min, and the ZOL was detected at 220 nm.

### pH-controlled release

Drug loaded ZZF@PVP nanoparticles (1 mg) were dispersed in 5 ml of 0.1 M PBS with pH values of 7.4 or 5.5 at 37 °C under gentle agitation. At regular time intervals, 200 μl of the dispersion was centrifuged at 12,000×*g* for 20 min over a period of 48 h, and the concentration of different drugs released into the supernatant was determined using the BCA kit (for BSA) and UV-Vis absorption (for DOX and siRNA).

### Preparation of cells

RAW264.7 cells (generously provided by Stem Cell Bank, Chinese Academy of Sciences) were used to investigate the biocompatibility and the intracellular behaviors of the nanoparticles. RAW 264.7 cells were maintained in DMEM containing 10% fetal bovine serum and 2 mM penicillin-streptomycin. Cell culture and experiments were performed under standard cell culture conditions of 37 °C, 5% CO_2_, and 95% humidity.

### In vitro cytotoxicity and cellular uptake of the ZZF@PVP nanoparticles

Cytotoxicity of the ZZF@PVP nanoparticles was determined by CCK-8 measurement as previously described. Briefly, RAW264.7 cells were seeded in a 96-well cell culture plate (1 × 104 cells/well). After being cultured for 12 h, the culture medium was replaced by a fresh medium containing different concentrations of ZZF@PVP nanoparticles. After incubation for an additional 24 h, the cell viability was determined by a CCK8 assay according to the instructions.

The cellular uptake of the ZZF@PVP nanoparticles was evaluated by confocal laser scanning microscopy (CLSM, LSM 900, ZEISS). First, fluorescein isothiocyanate (FITC) was encapsulated into nanoparticles through the abovementioned method, then RAW264.7 cells were seeded in a 15 mm cell culture plate (1^105^ cells/well). After being cultured for 12 h, the culture medium was replaced by a fresh medium containing 50 μg/ml of FITC@ZZF@PVP nanoparticles. After incubation for an additional 4 h, the cells were washed with PBS several times and incubated with DAPI for 30 min, the images were obtained using the CLSM.

### Animal model and in vivo bone target

All the reporting animal experiments followed the recommendations in the ARRIVE guidelines. Female Bal b/c mice were obtained from the Medical Experimental Animal Center of Central South University (Changsha, China), and all experiments involving animals were approved by the Ethics Committee of Xiang Ya Hospital, Central South University in China (201803106), and performed by the guidelines of the Department of Laboratory Animals.

To investigate the bone-targeting ability of the nanoparticles, a modified mouse calvaria resorption model^28^ was established. Before use, polyethylene particles were washed three times in 70% ethanol and oscillated for 48 h to remove endotoxins. After centrifugation, the PE particles were heated and dried at 40 °C. For surgery, a female Bal b/c mouse aged 6–7 weeks was anesthetized, then the skin was scrubbed twice using 75% ethanol. A 0.5 cm midline sagittal skin incision was made over the calvarium, then 15 mg of PE particles were uniformly placed over the periosteum between bregma and lambda with a sterile trimmed pipette. The incision was closed with a 4–0 suture (Ethicon, Somerville, USA). Sham controls underwent the same surgery procedures but without implanted PE particles. The wound healing of mice was observed, and the in vivo test was carried out two weeks after the operation.

Cy5.5 was encapsulated into ZIF-8 and ZZF@PVP nanoparticles through the method mentioned above as a tracer to detect the in vivo targeting ability of the nanoparticles. Then 2 mg nanoparticles were resuspended in 1 ml 0.1 M PBS (pH = 7.4) and the fluorescence intensity of Cy5.5 contained in both nanoparticles was measured by fluorescence spectrophotometer before injection. Mice through sham surgery were separated as the control group (n = 3) and mice after surgery with PE particles implanted were randomly divided into two groups (n = 5): ZIF-8 group and ZZF@PVP group. The different groups of mice were administered with 0.1 ml of Cy5.5@ZZF@PVP (for the control group and ZZF@PVP group) and Cy5.5@ZIF-8@PVP (for the ZIF-8 group) through the tail vein. Mice were anesthetized by gas inhalation using isoflurane, hair was removed from the surgical area, and fluorescence imaging was measured on an animal imaging system (IVIS Spectrum, PerkinElmer) at 6, 12, 24, and 48 h after injection. All mice were sacrificed on the 5th day and the organs of interest (heart, liver, spleen, lung, kidney, skull) collected for ex vivo fluorescence imaging. The images were acquired with a 660 nm excitation filter and a 710 nm emission filter, based on the detection of Cy5.5.

Main tissues (heart, liver, spleen, lung, and kidney) were collected for histopathology analysis. H&E-stained histological sections were observed via microscopy (Carl Zeiss Microscopy GmbH, Germany).

### Statistical analysis

All results were shown as mean values ± standard deviations. The statistical analysis was made by the Student's t-test using GraphPad Prism 5 software (GraphPad Software, Inc., La Jolla, USA). p < 0.05 was considered statistically significant.

## Conclusion

Our study explored a new and easy method of creating ZZF@PVP nanoparticles with high affinity to biological macromolecules, remarkable encapsulation ability for different kinds of materials, ability to release drugs in an acidic environment, and remarkable therapeutic potential for tumors, infections, and other acidic micro-environment lesions. Furthermore, this nano drug-delivery platform demonstrated excellent bone-targeting ability in a mouse calvaria resorption model. The potential of these bone-targeting nanoparticles in the treatment of bone disorders presents exciting scope for future research. Collectively, the results indicate that the ZZF@PVP nanoparticles nanoplatform is capable of carrying a variety of drugs, exhibiting controlled release in an acidic environment, and targeting the high bone turnover and destruction region effectively. The nanoplatform displays remarkable application prospects for bone-related diseases.

## Supplementary Information

Supplementary Information.

## Data Availability

The datasets used and/or analysed during the current study available from the corresponding author on reasonable request.
