# Supplementary material for: Zoledronate combined metal-organic frameworks for bone-targeting and drugs deliveries
Source: Sci Rep. 2022 Jul 19;12:12290. doi: 10.1038/s41598-022-15941-w (PMC9296467; doi:10.1038/s41598-022-15941-w)
Supplement: Supplementary file 1 — Supplementary Information. [file 41598_2022_15941_MOESM1_ESM.docx]

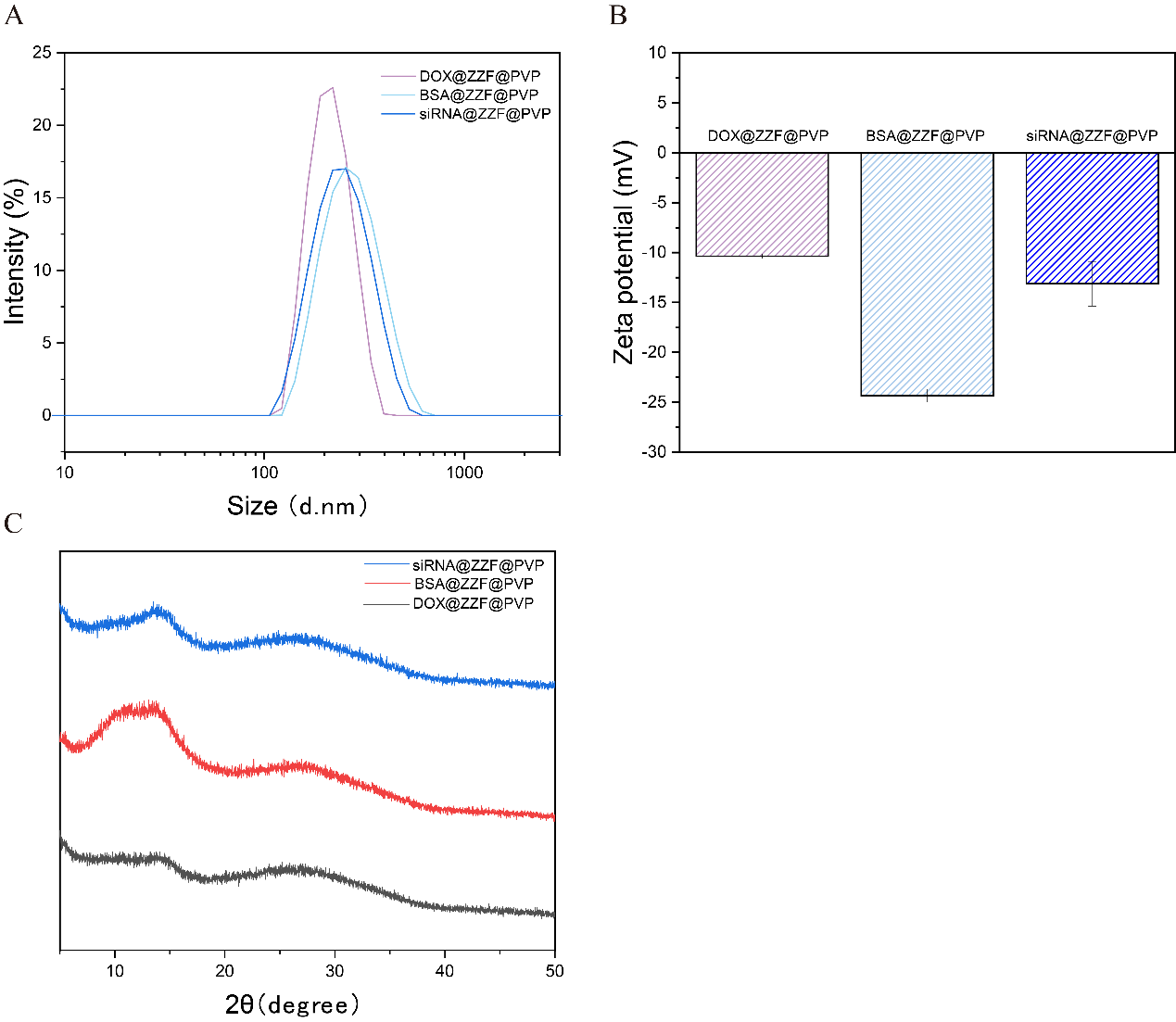


**Figure S1 (A) DLS, (B) zeta-potential, and (C) powder XRD of DOX@ZZF@PVP, BSA@ZZF@PVP, and siRNA@ZZF@PVP.**


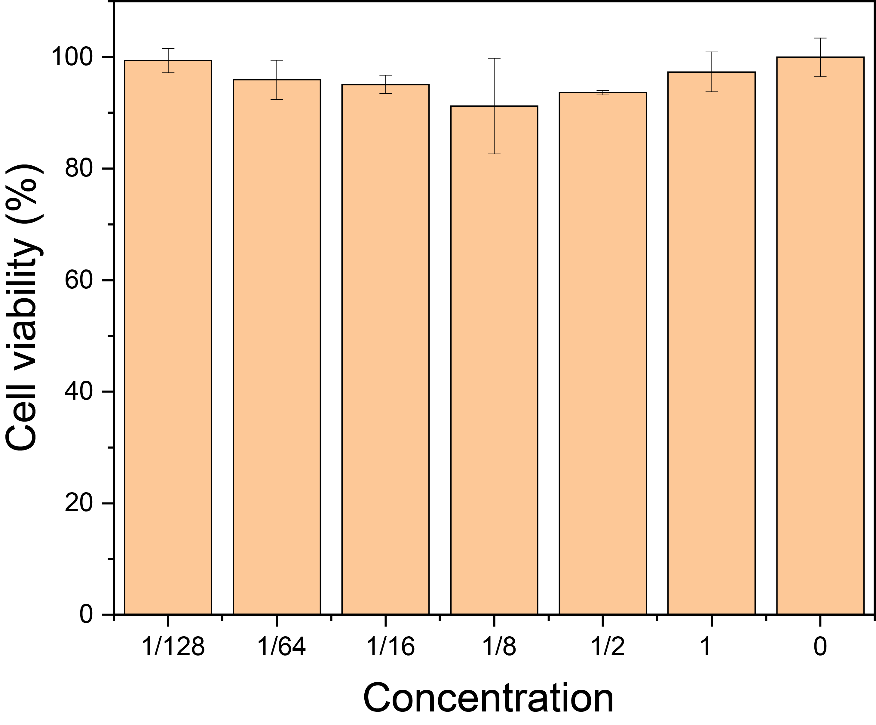


**Figure S2 The CCK-8 result of the ZOL on the RAW264.7 cells. Group 0 represent the control group. The concentration of ZOL in group 1 was 14.1μg/ mL, which simulated the concentration of ZOL released completely from 100μg/ml ZZF@PVP nanoparticles. The remaining groups represented the diluted in different multiples of group 1. After 48 h cell culture, the CCK-8 method was used to detect cell activity, and there was no statistical difference between groups and the control group.**
